# Supplementary material for: Extension of a multiphase tumour growth model to study nanoparticle delivery to solid tumours
Source: PLoS One. 2020 Feb 5;15(2):e0228443. doi: 10.1371/journal.pone.0228443 (PMC7001947; doi:10.1371/journal.pone.0228443)
Supplement: S1 Appendix — (PDF) [file pone.0228443.s001.pdf]

SUPPORTING INFORMATION

Extension of a multiphase tumour growth model to study nanoparticle delivery to solid tumours

Barbara Wirthl, Johannes Kremheller, Bernhard A. Schrefler, Wolfgang A. Wall

Nomenclature

Abbreviations

ALE	Arbitrary Lagrangian Eulerian
ECM	Extracellular matrix
EPR	Enhanced permeability and retention
IF	Interstitial fluid
LTC	Living tumour cells
NP	Nanoparticle
TAF	Tumour angiogenic factors
TCAT	Thermodynamically Constrained Averaging Theory

Configurations, domains and boundaries

Γ	Boundary of domain
Ω_0	Material/Reference configuration of 2D tissue domain
Ω_t	Current/Spatial configuration of 2D tissue domain

Computational solution approach

$\mathbf{R}$	Discrete nonlinear residual
n_{dim}	Number of spatial dimensions
n_{nodes}	Number of nodes of domain
n_{spec}	Number of species

Continuum mechanics

$\boldsymbol{\sigma}$	Cauchy stress tensor
$\boldsymbol{d}$	Displacement
$\boldsymbol{F}$	Deformation gradient
$\boldsymbol{S}$	Second Piola-Kirchhoff stress tensor
$\boldsymbol{X}$	Material/Reference coordinate
$\boldsymbol{v}^{\alpha}$	Velocity of phase α

J	Determinant of deformation gradient

Operators and symbols

$(\cdot)^T$	Transpose of a vector or matrix
$(\cdot)^{-1}$	Inverse of a matrix
$(\cdot)^{-T}$	Inverse of transpose of a matrix
$\mathbf{I}$, $\boldsymbol{I}$	Identity matrix
$\det$	Determinant of a matrix
$\boldsymbol{\nabla}\cdot$	Current divergence
$\boldsymbol{\nabla}_0\cdot$	Material divergence
$\boldsymbol{\nabla}$	Current gradient
$\boldsymbol{\nabla}_0$	Material gradient
$\langle\cdot\rangle_+$	Macaulay brackets
$H(\cdot)$	Heaviside function

Parameters of the tumour growth model

(S/V)	Surface-to-volume ratio
$\boldsymbol{k}^\alpha$	Isotropic permeability of the ECM with respect to a fluid phase α
μ^α	Viscosity of phase α
π^α	Oncotic pressure of phase α
ρ^α	Density of phase α
$\sigma_{\alpha\beta}$	Interfacial tension between phases α and β
D^v	Endothelial cell random-motility coefficient
$D^{i\alpha}$	Diffusion coefficient of species i in phase α
L_p	Hydraulic conductivity
P	Permeability
r	Radius
t	Vessel-wall thickness

Superscripts

α, β, κ	Arbitrary phase
h	Host cells
i	Arbitrary species
l	Interstitial fluid
ly	Lymph system

N	Necrotic tumour cells
n	Oxygen
s	Solid phase
t	Tumour cells
v	Vasculature

TCAT notation

$\mathfrak{I}_{c\alpha}$	Index set of entities connected to phase α
$\overset{\kappa\to\alpha}{M}$	Inter-phase mass transfer of from phase κ to phase α
$\overset{i\kappa\to i\alpha}{M}$	Inter-phase mass transfer of species i from phase κ to phase α
$\omega^{i\,\overline{\alpha}}$	Mass fraction of species i dissolved in phase α
$\sum_{\kappa\in\mathfrak{I}_{c\alpha}}$	Sum over all phases κ connected to phase α
$\varepsilon^{\alpha}r^{i\alpha}$	Intra-phase reaction term of species i in phase α

Variables of the tumour growth model

ε	Volume fraction of pores of the ECM
ε^{α}	Volume fraction of phase α
ψ^{α}	Generic primary variable of phase $\alpha = t, h, l$
p^{α}	Pressure of phase α
$p^{\alpha\beta}$	Differential pressure between phase α and β
$P^{\alpha}_{\mathrm{oxy}}$	Oxygen partial pressure in phase α
S^{α}	Saturation of phase $\alpha = t, h, l$

A.1 The vascular multiphase tumour growth model

Here, we summarise all additional model equations for the sake of completeness. The volume fraction of the porous network filled by host cells, tumour cells and IF is derived from the balance of mass of the solid phase in the reference configuration, as described in Kremheller et al. [1] and reads

$$\varepsilon = 1 - \varepsilon^v - \frac{1 - \varepsilon_0 - \varepsilon_0^v}{J} \tag{A.1}$$

with the Jacobian of the deformation gradient J and the initial volume fractions ε_0 and ε_0^v. We use the same saturation-pressure relationship $S^\alpha\left(p^{\alpha\beta}\right)$ as presented in Sciumè et al. [2] with a pressure difference $p^{\alpha\beta}$ between phases α and β

$$p^{hl}\left(S^l\right) = p^h - p^l = a \cdot \tan\left[\frac{\pi}{2}\left(1 - S^l\right)^b\right] \tag{A.2}$$

$$p^{th}\left(S^t\right) = p^t - p^h = a\frac{\sigma_{th}}{\sigma_{hl}} \cdot \tan\left[\frac{\pi}{2}\left(S^t\right)^b\right] \tag{A.3}$$

with the interfacial tension between the fluids $\sigma_{\alpha\beta}$ and model constants a and b. For host cells, the relation is given by

$$S^h\left(p^{th}, p^{hl}\right) = 1 - S^l\left(p^{hl}\right) - S^t\left(p^{th}\right). \tag{A.4}$$

A.1.1 Solid phase

We model the ECM as a porous solid phase which represents the skeleton of the porous system. Based on Terzaghi's effective stress principle [3], the effective stress $\boldsymbol{\sigma}_{\text{eff}}$ is defined as the sum of the total stress $\boldsymbol{\sigma}_{\text{tot}}$ in the solid phase and the solid pressure p^s

$$\boldsymbol{\sigma}^s_{\text{eff}} = \boldsymbol{\sigma}^s_{\text{tot}} + p^s\boldsymbol{I}. \tag{A.5}$$

This equation demonstrates the interaction between solid and fluid phases in a porous system. The solid pressure p^s is defined as the weighted sum of fluid pressures p^α and saturations S^α and the blood pressure p^v in the vasculature

$$p^s = \frac{\varepsilon}{\varepsilon + \varepsilon^v} \sum_{\alpha = t,h,l} S^\alpha p^\alpha + \frac{\varepsilon^v}{\varepsilon + \varepsilon^v} p^v \tag{A.6}$$

with the volume fraction ε of host cells, tumour cells and IF

$$\varepsilon = \varepsilon^h + \varepsilon^t + \varepsilon^l. \tag{A.7}$$

In the absence of body and dynamic forces, the ECM is governed by the balance of momentum pulled back into material configuration

$$\boldsymbol{\nabla}_0 \cdot \left(\boldsymbol{F} \cdot \boldsymbol{S}^s_{\text{eff}} - \boldsymbol{F} \cdot J\boldsymbol{F}^{-1} \cdot \boldsymbol{F}^{-T} p^s\right) = \boldsymbol{0} \tag{A.8}$$

where $\boldsymbol{\nabla}_0$ denotes the material divergence operator. $\boldsymbol{F}$ denotes the deformation gradient and J its determinant. $\boldsymbol{S}$ denotes the second Piola-Kirchhoff stress.

A.1.2 Tumour cells, host cells and the IF

The pore space of the ECM is occupied by three fluid phases and a further porous network. The three fluid phases are the tumour cells, host cells and the IF while the additional porous network is the vasculature. The saturations of tumour cells, host cells and the IF S^α are defined as

$$S^\alpha = \frac{\varepsilon^\alpha}{\varepsilon}, \quad \text{where} \quad \alpha = h, t, l \tag{A.9}$$

Table A.1: **Mass transfer terms for the different phases of the tumour growth model.** The ECM represents the solid phase. Host cells, tumour cells and the IF are modelled as fluid phases occupying the pores of the ECM together with the vasculature, which is included as additional porous network. Details on the mass transfer terms are given in Sec A.1.5.

Entity	Symbol		Term
Extracellular Matrix (ECM)	$\sum_{\kappa \in \mathcal{J}_{cs}} \overset{\kappa\to s}{M}$	=	0
Host cells	$\sum_{\kappa \in \mathcal{J}_{ch}} \overset{\kappa\to h}{M}$	=	0
Tumour cells	$\sum_{\kappa \in \mathcal{J}_{ct}} \overset{\kappa\to t}{M}$	=	$\overset{l\to t}{M}_{\text{growth}}$
Interstitial fluid (IF)	$\sum_{\kappa \in \mathcal{J}_{cl}} \overset{\kappa\to l}{M}$	=	$-\overset{l\to t}{M}_{\text{growth}} + \overset{v\to l}{M}_{\text{leak}} - \overset{l\to ly}{M}_{\text{drain}}$

which directly leads to

$$\sum_{\alpha} S^{\alpha} = 1, \quad \text{where} \quad \alpha = h, t, l. \tag{A.10}$$

Tumour cells, host cells and IF are modelled as fluid phases [4]. The mass balance equation of host cells and tumour cells ($\alpha = h, t$) is given by

$$\begin{aligned} &\varepsilon \sum_{\beta=t,h,l} \frac{\partial S^{\alpha}}{\partial \psi^{\beta}} \frac{\partial \psi^{\beta}}{\partial t}\bigg|_{\boldsymbol{X}} - S^{\alpha} \frac{\partial \varepsilon^{v}}{\partial t}\bigg|_{\boldsymbol{X}} + S^{\alpha}(1-\varepsilon^{v}) \boldsymbol{\nabla} \cdot \boldsymbol{v}^{s} - \boldsymbol{\nabla} \cdot \left(\frac{\boldsymbol{k}^{\alpha}}{\mu^{\alpha}} \sum_{\beta=t,h,l} \frac{\partial p^{\alpha}}{\partial \psi^{\beta}} \boldsymbol{\nabla} \psi^{\beta} \right) \\ &\qquad = \frac{\sum_{\kappa \in \mathcal{J}_{c\alpha}} \overset{\kappa\to\alpha}{M}}{\rho^{\alpha}} + S^{\alpha} \frac{\sum_{\kappa \in \mathcal{J}_{cs}} \overset{\kappa\to s}{M}}{\rho^{s}} \quad \text{in } \Omega_t \times [t_0, t_E] \end{aligned} \tag{A.11}$$

and of the IF ($\alpha = l$)

$$\begin{aligned} &-\frac{\partial \varepsilon^{v}}{\partial t}\bigg|_{\boldsymbol{X}} + (1-\varepsilon^{v}) \boldsymbol{\nabla} \cdot \boldsymbol{v}^{s} - \sum_{\gamma=t,h,l} \left(\boldsymbol{\nabla} \cdot \left(\frac{\boldsymbol{k}^{\gamma}}{\mu^{\gamma}} \sum_{\beta=t,h,l} \frac{\partial p^{\gamma}}{\partial \psi^{\beta}} \boldsymbol{\nabla} \psi^{\beta} \right) \right) \\ &\qquad = \sum_{\gamma=t,h,l} \left(\frac{\sum_{\kappa \in \mathcal{J}_{c\gamma}} \overset{\kappa\to\gamma}{M}}{\rho^{\gamma}} \right) + \frac{\sum_{\kappa \in \mathcal{J}_{cs}} \overset{\kappa\to s}{M}}{\rho^{s}} \quad \text{in } \Omega_t \times [t_0, t_E] \end{aligned} \tag{A.12}$$

which are equivalent to the equations derived in Kremheller et al. [1]. Herein, μ^{α} denotes the viscosity and $\boldsymbol{v}^{s}$ the velocity of the ECM. The isotropic permeability of the ECM with respect to a fluid phase α is defined as

$$\boldsymbol{k}^{\alpha} = (S^{\alpha})^{A_{\alpha}} \cdot k \cdot \boldsymbol{I}, \quad \text{where} \quad \alpha = h, t, l \tag{A.13}$$

whereby we employ the power law presented in Sciumè et al. [2] with $A_{\alpha} > 1$, the intrinsic permeability of the ECM k and the unit tensor $\boldsymbol{I}$. Each primary variable ψ^{α} is either chosen as the saturation S^{α}, pressure p^{α} or differential pressure $p^{\alpha\beta} = p^{\alpha} - p^{\beta}$. The derivation of these equations is presented in Kremheller et al. [1] and based on earlier work by Sciumè et al. [4, 2, 5]. The equations are written in Arbitrary Lagrangian Eulerian (ALE) form with spatial configuration Ω_t. The considered time interval is $[t_0, t_E]$.

The mass transfer terms $\overset{\kappa\to\alpha}{M}$ for the different phases are summed up in Table A.1. In accordance with the standard TCAT notation as presented in Gray and Miller [6], $\mathcal{J}_{c\alpha}$ denotes the connected set of phases and common curves to interface α, i.e. all interfaces that separate phase α from adjacent phases.

A.1.3 Vasculature

We treat the vasculature in a homogenised way as an additional pore space in the ECM [1]. The volume fraction of perfused vessels ε^v follows an evolution equation

$$\left.\frac{\partial \varepsilon^v}{\partial t}\right|_{\boldsymbol{X}} + \varepsilon^v\, \boldsymbol{\nabla} \cdot \boldsymbol{v}^s - \boldsymbol{\nabla} \cdot (D^v\, \boldsymbol{\nabla} \varepsilon^v) = -M_{\text{coll}} \tag{A.14}$$

with D^v being the endothelial cell random-motility coefficient [7]. A growing tumour develops elevated solid stresses which deform surrounding tissues and compress or even collapse blood and lymphatic vessels [8]. Based on the work of Padera et al. [9], who state that proliferating tumour cells cause the collapse of intratumour vessels, we use a simple model to include blood-vessel collapse. We assume that a tumour pressure p^t above p^t_{coll} causes the vasculature to collapse according to a Gompertz function given by

$$M_{\text{coll}} = \gamma^v_{\text{coll}} \exp\left[-\exp\left(-\mathcal{A} \cdot \frac{p^t - p^v_{\text{coll}}}{\mathcal{B}}\right)\right] H(\varepsilon^v) \tag{A.15}$$

with $\gamma^v_{\text{coll}} = 3.0 \times 10^{-11}\,\text{s}^{-1}$, $p^v_{\text{coll}} = 1000\,\text{Pa}$, $\mathcal{A} = 40$ and $\mathcal{B} = 1000\,\text{Pa}$ and $H(\cdot)$ denoting the Heaviside function.

This formulation includes several simplifications and assumptions. First, we do not study blood flow and species transport in the vasculature. Our focus is on the nanoparticle transport from the blood vessels to the tumour and not on the transport in the vessels themselves. We therefore assume the blood pressure to be constant, $p^v = 20\,\text{mmHg}$, along with all mass fractions of species in the vasculature. We further use the continuum homogenised approach [1] because the quantities of interest characterising blood flow and species transport are macroscopic and averaged in the sense of TCAT [6, 4]; in contrast, a discrete or a hybrid model [7, 10] captures locally resolved processes. Second, we neglect angiogenesis and hence the growth of new blood vessels. The results of Kremheller et al. [1, 10] show that angiogenesis only has a minor influence on tumour growth during the first six days. We therefore neglect the formation of new blood vessels on this timescale. Nevertheless, we could easily include chemotaxis in response to tumour angiogenic factor (TAF) gradients which we introduced in Kremheller et al. [1]. Third, we use a simple heuristic model for blood-vessel collapse and choose the necessary parameters in such a way that the grown tumour contains a non-perfused inner core. Studying the intricate interplay between angiogenesis and blood-vessel collapse would be beyond the scope of this work, but could easily be added through suitable models.

A.1.4 Species transport: Oxygen and necrotic tumour cells

Each phase may contain several species whose transport is governed by convection and diffusion. The mass balance equation for a species i with mass fraction $\omega^{i\bar{\alpha}}$ in phase α is given by

$$\begin{aligned} \varepsilon S^\alpha \left.\frac{\partial \omega^{i\bar{\alpha}}}{\partial t}\right|_{\boldsymbol{X}} - \frac{\boldsymbol{k}^\alpha}{\mu^\alpha} \boldsymbol{\nabla} p^\alpha \cdot \boldsymbol{\nabla} \omega^{i\bar{\alpha}} - \boldsymbol{\nabla} \cdot \left(\varepsilon S^\alpha D^{i\alpha}_{\text{eff}} \boldsymbol{\nabla} \omega^{i\bar{\alpha}}\right) \\ = \frac{1}{\rho^\alpha} \left(\sum_{\kappa \in \mathcal{J}_{c\alpha}} \overset{i\kappa \to i\alpha}{M} + \varepsilon^\alpha r^{i\alpha} - \omega^{i\bar{\alpha}} \sum_{\kappa \in \mathcal{J}_{c\alpha}} \overset{\kappa \to \alpha}{M} \right) \end{aligned} \tag{A.16}$$

with the effective diffusivity $D^{i\alpha}_{\text{eff}}$. The terms on the right-hand side of the equation describe mass transfer of species between the different phases. The first term on the right-hand side of the equation, $\overset{i\kappa \to i\alpha}{M}$, denotes mass transfer of species i from all phases κ to the considered phase α. The second term is an intra-phase reaction term, e.g. a source or sink term for species i. The third term results from applying the product rule in the mass balance of species. For details on the derivation of Equation (A.16) see Sciumè et al. [4].

We consider two species: the necrotic tumour cells (N) and oxygen (n). The necrotic tumour cells $\omega^{N\bar{t}}$ are a portion of the tumour cells t. Oxygen $\omega^{n\bar{l}}$ is the only nutrient in the current model and

Table A.2: **Mass transfer terms for species.** Necrotic tumour cells are the necrotic portion of the tumour cells. Oxygen and nanoparticles are dissolved in the IF.

**Species**	**Phase**	**Term on the right-hand side of Eq (A.16)**
Necrotic tumour cells	Tumour cells	$\frac{1}{\rho^t}\left(\varepsilon^t r^{Nt} - \omega^{N\bar{t}} \overset{l\to t}{M}_{\text{growth}}\right)$
Oxygen	IF	$\frac{1}{\rho^l}\left(-\overset{nl\to t}{M}_{\text{cons}} - \overset{nl\to h}{M}_{\text{cons}} + \overset{nv\to nl}{M}_{\text{tc}} + \omega^{n\bar{l}} \overset{l\to t}{M}_{\text{growth}}\right)$

is transported by the IF. In Kremheller et al. [10], we have introduced blood flow in the vasculature and species transport therein. For simplicity, we here assume a constant mass fraction of oxygen in the vasculature. As oxygen is the only nutrient considered, it governs and limits the growth of the tumour. The transfer terms for necrotic tumour cells and oxygen are summarised in Table A.2 and in Section A.1.5.

A.1.5 Mass transfer equations

Leakage of fluid from the vasculature to the IF due to abnormal vessel characteristics is given by

$$\overset{v\to l}{M}_{\text{leak}} = \rho^v \cdot L_p^v \cdot \frac{S}{V} \cdot \left\langle p^{\text{eff}} - p^l \right\rangle_+ \cdot \varepsilon^v, \tag{A.17}$$

where a net outflow from the vessel into the IF is generated if the effective pressure

$$p^{\text{eff}} = p^v - \sigma\left(\pi^v - \pi^l\right) \tag{A.18}$$

is higher than the interstitial pressure p^l. We employ a Starling equation with hydraulic conductivity of the membrane L_p and surface-to-volume ratio S/V which describes transendothelial fluid exchange in capillaries. The plasma protein oncotic pressure is denoted as π^v and the interstitial oncotic pressure as π^l. σ is the Staverman's reflection coefficient. The hydraulic conductivity L_p^v is estimated based on Eq (6) as $L_p^v = 1.25 \times 10^{-6}\,\text{mm}/(\text{Pa s})$ with the fraction of pores $\gamma_p = 10 \times 10^{-4}$ [11], the thickness of the vessel wall $t = 1\,\text{nm}$ [12] and the pore radius $r_0 = 200\,\text{nm}$ [13]. We further incorporate uptake of IF by the lymph system in the form of

$$\overset{l\to ly}{M}_{\text{drain}} = \rho^l \cdot \left(L_p \frac{S}{V}\right)^{ly} \cdot \left\langle p^l - p^{\text{ly}} \right\rangle_+ \cdot \left\langle 1 - \frac{p^t}{p^{ly}_{\text{coll}}} \right\rangle_+ \tag{A.19}$$

with $\left(L_p \cdot \frac{S}{V}\right)^{ly} = 1.04 \times 10^{-6}\,(\text{Pa s})^{-1}$ being the lymphatic filtration coefficient as given by Baxter and Jain [14]. We employ the values for L_p^v and $\left(L_p \cdot \frac{S}{V}\right)^{ly}$ for the tumour growth simulation in Section 3.1. For the subsequent nanoparticle transport study in Section 3.2, we only vary the mass transfer terms for nanoparticles given by Eq (4). We do not change L_p^v and $\left(L_p \cdot \frac{S}{V}\right)^{ly}$ in $\overset{v\to l}{M}_{\text{leak}}$ and $\overset{l\to ly}{M}_{\text{drain}}$ (see Table A.1), respectively, to ensure a uniform initial condition for all transport simulations, and to avoid the latter being influenced by a change in IF pressure gradient.

Growth of the tumour phase is described as

$$\overset{l\to t}{M}_{\text{growth}} = \left(\gamma^t_{\text{growth}} \left\langle \frac{\omega^{n\bar{l}} - \omega^{n\bar{l}}_{\text{crit}}}{\omega^{n\bar{l}}_{\text{env}} - \omega^{n\bar{l}}_{\text{crit}}} \right\rangle_+\right) \left(1 - \omega^{N\bar{t}}\right) \varepsilon S^t \tag{A.20}$$

where γ^t_{growth} denotes the nutrient uptake and consumption of water necessary for cell growth from IF, and $\omega^{n\bar{l}}_{\text{env}}$ the environmental mass fraction of oxygen, e.g. in healthy tissue further away from

the tumour. When the mass fraction of oxygen $\omega^{n\bar{l}}$ falls below the critical value $\omega^{n\bar{l}}_{\text{crit}}$, cell growth is inhibited. The only nutrient which is explicitly included in our model is oxygen. The growing tumour consumes oxygen from the IF modelled as

$$\overset{nl\to t}{M}_{\text{cons}} = \overset{nl\to nt}{M} = \left(\gamma^{nt}_{\text{growth}} \left\langle \frac{\omega^{n\bar{l}} - \omega^{n\bar{l}}_{\text{crit}}}{\omega^{n\bar{l}}_{\text{env}} - \omega^{n\bar{l}}_{\text{crit}}} \right\rangle_{+} + \gamma^{nt}_{0} \sin\left(\frac{\pi}{2}\frac{\omega^{n\bar{l}}}{\omega^{n\bar{l}}_{\text{env}}}\right)\right) \left(1 - \omega^{N\bar{t}}\right) \varepsilon S^{t} \tag{A.21}$$

with coefficients $\gamma^{nt}_{\text{growth}}$ and γ^{nt}_{0} describing oxygen consumption related to growth and to normal metabolism, respectively [2]. Oxygen consumption by host cells as described in Kremheller et al. [10] is given by

$$\overset{nl\to h}{M}_{\text{cons}} = \overset{nl\to nh}{M} = \gamma^{nh}_{0} \cdot \sin\left(\frac{\pi}{2}\frac{\omega^{n\bar{l}}}{\omega^{n\bar{l}}_{\text{env}}}\right) \cdot \varepsilon S^{h} \tag{A.22}$$

where γ^{nh}_{0} models the oxygen demand of host cells. Transcapillary exchange of oxygen is written as

$$\overset{nv\to nl}{M}_{\text{tv}} = \gamma_{\text{tv}} \cdot \rho^{n} \cdot \left(\frac{S}{V}\right) \cdot \left\langle P^{v}_{\text{oxy}} - P^{l}_{\text{oxy}} \right\rangle_{+} \cdot \varepsilon^{v} \tag{A.23}$$

based on Kremheller et al. [10] The mass fraction of oxygen in the IF $\omega^{n\bar{l}}$ and the vasculature $\omega^{n\bar{v}}$ depend on the oxygen partial pressures P^{l}_{oxy} and P^{v}_{oxy} in the IF and the vasculature, respectively, as follows

$$\omega^{n\bar{l}}\left(P^{l}_{\text{oxy}}\right) = \frac{\rho^{n}}{\rho^{l}} \cdot \alpha_{l} P^{l}_{\text{oxy}}, \tag{A.24}$$

$$\omega^{n\bar{v}}\left(P^{v}_{\text{oxy}}\right) = \frac{\rho^{n}}{\rho^{v}} \cdot \left(\alpha_{v,\text{eff}} P^{v}_{\text{oxy}} + H_{D} \cdot C^{n\bar{v}}_{0} \cdot S\left(P^{v}_{\text{oxy}}\right)\right) \tag{A.25}$$

with the effective solubility $\alpha_{v,\text{eff}}$, the discharge hematocrit H_D and the concentration of oxygen at maximum saturation $C^{n\bar{v}}_{0}$. We further apply the Hill equation for the binding of oxygen to haemoglobin

$$S\left(P^{v}_{\text{oxy}}\right) = \frac{\left(P^{v}_{\text{oxy}}\right)^{n}}{\left(P^{v}_{\text{oxy}}\right)^{n} + \left(P^{v}_{\text{oxy},50}\right)^{n}} \tag{A.26}$$

with the Hill exponent n and the partial pressure at 50% oxygen saturation $P^{v}_{\text{oxy},50}$. A power relationship [2, 5] is used for the effective diffusivity D^{nl}_{eff} of oxygen in the IF

$$D^{nl}_{\text{eff}} = D^{nl}_{0} \left(\varepsilon S^{l}\right)^{\delta}. \tag{A.27}$$

The death rate of tumour cells due to hypoxia or nutrient shortage is given by

$$\varepsilon^{t} r^{Nt} = \gamma^{t}_{\text{necrosis}} \left\langle \frac{\omega^{n\bar{l}}_{\text{crit}} - \omega^{n\bar{l}}}{\omega^{n\bar{l}}_{\text{env}} - \omega^{n\bar{l}}_{\text{crit}}} \right\rangle_{+} \left(1 - \omega^{N\bar{t}}\right) \varepsilon S^{t} \tag{A.28}$$

with the necrosis coefficient $\gamma^{t}_{\text{necrosis}}$ regulating the rate of cell death.

A.1.6 Numerical solution

From a computational point of view, the model described above leads to a strongly coupled problem consisting of three fields: the structure field with displacements $\mathbf{d}^{s}$ as primary variables; the fluid field with user specific fluid primary variables $\boldsymbol{\psi}^{t,h,l}$; and species transport with species mass fractions $\boldsymbol{\omega}$ as primary variables. The fluid primary variables can be selected from saturations S, pressures p or differential pressures $p^{\alpha\beta}$. The weak form of the governing equations is obtained by employing the standard Galerkin procedure. Thereafter, the equations are discretised in space using finite elements [15] and in time using the one-step-theta scheme [16].

The weak form of the governing equations discretised in time and space is written as discrete nonlinear residual $\mathbf{R}$ at time step $n+1$

$$\mathbf{R}^s_{n+1}\left(\underline{\mathbf{d}^s_{n+1}}, \boldsymbol{\psi}^{t,h,l}_{n+1}, \boldsymbol{\varepsilon}^v_{n+1}\right) = \mathbf{0} \quad \text{where} \quad \mathbf{R}^s_{n+1} \in \mathbb{R}^{n_{\text{nodes}} \cdot n_{\text{dim}}} \tag{A.29}$$

$$\mathbf{R}^{t,h,l,v}_{n+1}\left(\mathbf{d}^s_{n+1}, \underline{\boldsymbol{\psi}^{t,h,l}_{n+1}}, \underline{\boldsymbol{\varepsilon}^v_{n+1}}, \boldsymbol{\omega}_{n+1}\right) = \mathbf{0} \quad \text{where} \quad \mathbf{R}^{t,h,l,v}_{n+1} \in \mathbb{R}^{n_{\text{nodes}} \cdot 4} \tag{A.30}$$

$$\mathbf{R}^{\text{spec}}\left(\mathbf{d}^s_{n+1}, \boldsymbol{\psi}^{t,h,l}_{n+1}, \boldsymbol{\varepsilon}^v_{n+1}, \underline{\boldsymbol{\omega}_{n+1}}\right) = \mathbf{0} \quad \text{where} \quad \mathbf{R}^{\text{spec}}_{n+1} \in \mathbb{R}^{n_{\text{nodes}} \cdot n_{\text{spec}}} \tag{A.31}$$

with n_{nodes} being the number of nodes, n_{dim} the number of spatial dimensions and n_{spec} the number of species in the multiphase model. The underlined terms mark the primary variables of the respective equations. The first residual $\mathbf{R}^s$ describes the discrete residual of the solid phase. The second residual $\mathbf{R}^{t,h,l,v}$ includes the discrete residuals of tumour cells, host cells, IF as well as of the vasculature. The third residual $\mathbf{R}^{\text{spec}}$ is the discrete residual of all species in the multiphase system. We choose the primary variables of the fluid phases and the species as follows

$$\boldsymbol{\psi}^{t,h,l} = \left[\, p^{hl},\, p^{th},\, p^{l} \,\right] \tag{A.32}$$

$$\boldsymbol{\omega} = \left[\, \omega^{n\bar{l}},\, \omega^{N\bar{t}},\, \omega^{n\bar{v}},\, \omega^{\text{NP}\bar{v}},\, \omega^{\text{NP}\bar{l}} \,\right]. \tag{A.33}$$

This results in a nonlinear coupled problem with three fields which we solve simultaneously with a monolithic scheme, as presented in Kremheller et al. [1]. For the implementation and for all simulations, we use BACI - an in-house parallel multiphysics research code developed at the Institute for Computational Mechanics of the Technical University of Munich [17].

A.2 Additional parameters

Table A.3: Parameters of host cells, tumour cells and IF which are modelled as fluid phases governed by Eq (A.11) and (A.12).

Symbol	Parameter	Value	Units	Source
ε_0	Initial volume fraction of HC, TC and IF	0.78	-	[1]
ρ^h, ρ^t, ρ^l	Density of fluid phases	1.0×10^{-3}	$\mathrm{g/mm^3}$	[2]
μ^h, μ^t	Dynamic viscosity of host cells and tumour cells	20	$\mathrm{Pa\,s}$	[5]
μ^l	Dynamic viscosity of IF	1.0×10^{-3}	$\mathrm{Pa\,s}$	[2]
A_h, A_t	Exponent in Eq (A.13) for host cells and tumour cells	2	-	[2]
A_l	Exponent in Eq (A.13) for IF	4	-	[1]
σ_{hl}	Host cells-IF interfacial tension	72	$\mathrm{g/s^2}$	[2]
σ_{th}	Tumour cells-host cells interfacial tension	36	$\mathrm{g/s^2}$	[2]
a	Coefficient in Eq (A.2) and (A.3)	590	Pa	[2]
b	Coefficient in Eq (A.2) and (A.3)	1	-	[2]
$\gamma^t_{\mathrm{growth}}$	Growth coefficient of tumour cells	4.0×10^{-8}	$\mathrm{g/(mm^3\,s)}$	[5]
$\left(L_p \cdot \frac{S}{V}\right)^{ly}$ (⋆)	Lymphatic filtration coefficient	1.04×10^{-6}	$\mathrm{1/(Pa\,s)}$	[14]
p^{ly}	Lymphatic pressure	0	mmHg	[1]
p^{ly}_{coll}	Threshold for lymphatic vessel collapse	1000	Pa	

(⋆) We use this value for the tumour growth simulation in Section 3.1 as well as for $\overset{l\to ly}{M}_{\mathrm{drain}}$ in the transport study in Section 3.2.

Table A.4: Parameters of the vasculature and for leakage from the vasculature to the IF.

Symbol	Parameter	Value	Units	Source
ρ^v	Density of blood	1.0×10^{-3}	$\mathrm{g/mm^3}$	[10]
p^v	Blood pressure	20	mmHg	
μ^v	Dynamic viscosity of blood	4.0×10^{-3}	$\mathrm{Pa\,s}$	[18]
D^v	Endothelial cell random-motility coefficient	5.0×10^{-9}	$\mathrm{mm^2/s}$	[1]
$\sigma(\pi^v - \pi^l)$	Osmotic pressure difference	1333	Pa	[19]
L^v_p (⋆)	Hydraulic conductivity for transcapillary flow	0.7×10^{-6}	$\mathrm{mm/(Pa\,s)}$	[11]
S/V	Surface-to-volume ratio	20	$\mathrm{1/mm}$	[20]

(⋆) We use this value for the tumour growth simulation in Section 3.1 as well as for $\overset{v\to l}{M}_{\mathrm{leak}}$ in the transport study in Section 3.2.

Table A.5: Parameters for transport of necrotic tumour cells and oxygen governed by Eq (A.16).

Symbol	Parameter	Value	Units	Source
D_0^{nl}	Interstitial diffusivity of oxygen	3.2×10^{-3}	$\mathrm{mm^2/s}$	[2]
δ	Nonlinear diffusion law of oxygen	2	-	[2]
D_{eff}^{Nt}	Diffusion coefficient of necrotic tumour cells	0	$\mathrm{mm^2/s}$	[1]
$\gamma_{\mathrm{necrosis}}^{t}$	Necrosis coefficient	1.0×10^{-8}	$\mathrm{g/(mm^3\,s)}$	[21]
$\gamma_{\mathrm{growth}}^{nt}$ $^{(\star)}$	Consumption related to growth	2.4×10^{-10}	$\mathrm{g/(mm^3\,s)}$	[2]
γ_0^{nt} $^{(\star)}$	Consumption due to metabolism of tumour cells	6.0×10^{-10}	$\mathrm{g/(mm^3\,s)}$	[2]
γ_0^{nh}	Consumption due to metabolism of host cells	2.0×10^{-10}	$\mathrm{g/(mm^3\,s)}$	[10]
$\omega_{\mathrm{crit}}^{n\bar{l}}$	Critical mass fraction of oxygen	1.0×10^{-6}	-	[5]
$\omega_{\mathrm{env}}^{n\bar{l}}$	Environmental mass fraction of oxygen	4.2×10^{-6}	-	[2]

$^{(\star)}$ These parameters have been previously denoted by $\gamma_{\mathrm{growth}}^{nl}$ and γ_0^{nl}, respectively [2].

Table A.6: Parameters for oxygen exchange governed by Eq (A.23) - (A.26) as presented in Kremheller *et al.* [10].

Symbol	Parameter	Value	Units	Source
ρ^n	Density of oxygen	1.429×10^{-6}	$\mathrm{g/mm^3}$	[10]
α_l	Solubility of oxygen in the IF	3.0×10^{-5}	$\mathrm{mmHg^{-1}}$	[22]
$\alpha_{v,\mathrm{eff}}$	Effective solubility of oxygen in blood	3.1×10^{-5}	$\mathrm{mmHg^{-1}}$	[23]
H_D	Discharge hematocrit	0.45	-	[23]
$C_0^{n\bar{v}}$	Concentration of oxygen at maximum saturation	0.5	-	[23]
n	Hill exponent	2.7	-	[24]
$P_{\mathrm{oxy},50}^{v}$	Partial pressure at 50% oxygen saturation	37	mmHg	[24]
γ_{tv}	Coefficient for transvascular oxygen exchange	1.429×10^{-5}	$\mathrm{mm/(mmHg\,s)}$	[23]

Table A.7: Parameters of the ECM as solid phase modelled with a Neo-Hookean material law.

Symbol	Parameter	Value	Units	Source
ρ^s	Density of ECM	1×10^{-3}	$\mathrm{g/mm^3}$	[2]
ν	Poisson's ratio of ECM	0.4	-	[2]
E	Young's modulus of ECM	800	Pa	[1]
k	Intrinsic permeability of ECM	1×10^{-9}	$\mathrm{mm^2}$	[1]

References

[1] J. Kremheller, A.-T. Vuong, L. Yoshihara, W. A. Wall, and B. A. Schrefler. “A monolithic multiphase porous medium framework for (a-)vascular tumor growth”. In: *Computer Methods in Applied Mechanics and Engineering* 340 (2018), pp. 657–683. DOI: 10.1016/j.cma.2018.06.009.

[2] G. Sciumè, W. G. Gray, F. Hussain, M. Ferrari, P. Decuzzi, and B. A. Schrefler. “Three phase flow dynamics in tumor growth”. In: *Computational Mechanics* 53.3 (2014), pp. 465–484. DOI: 10.1007/s00466-013-0956-2.

[3] O. Coussy. *Thermodynamics*. John Wiley & Sons, 2003. DOI: doi:10.1002/0470092718.ch3.

[4] G. Sciumè, S. Shelton, W. G. Gray, C. T. Miller, F. Hussain, M. Ferrari, P. Decuzzi, and B. A. Schrefler. “A multiphase model for three-dimensional tumor growth”. In: *New Journal of Physics* 15 (2013). DOI: 10.1088/1367-2630/15/1/015005.

[5] G. Sciumè, R. Santagiuliana, M. Ferrari, P. Decuzzi, and B. A. Schrefler. “A tumor growth model with deformable ECM”. In: *Physical Biology* 11.6 (2014). DOI: 10.1088/1478-3975/11/6/065004.

[6] W. G. Gray and C. T. Miller. *Introduction to the Thermodynamically Constrained Averaging Theory for Porous Medium Systems*. Cham: Advances in Geophysical, Environmental Mechanics, and Mathematics. Springer International Publishing, 2014. DOI: 10.1007/978-3-319-04010-3.

[7] A. R. Anderson and M. A. Chaplain. “Continuous and discrete mathematical models of tumor-induced angiogenesis”. In: *Bulletin of Mathematical Biology* 60.5 (1998), pp. 857–899. DOI: 10.1006/bulm.1998.0042.

[8] T. Stylianopoulos, J. D. Martin, V. P. Chauhan, S. R. Jain, B. Diop-Frimpong, N. Bardeesy, B. L. Smith, C. R. Ferrone, F. J. Hornicek, Y. Boucher, L. L. Munn, and R. K. Jain. “Causes, consequences, and remedies for growth-induced solid stress in murine and human tumors”. In: *Proceedings of the National Academy of Sciences* 109.38 (2012), pp. 15101–15108. DOI: 10.1073/pnas.1213353109.

[9] T. P. Padera, B. R. Stoll, J. B. Tooredman, D. Capen, E. di Tomaso, and R. K. Jain. “Cancer cells compress intratumour vessels”. In: *Nature* 427.6976 (2004), pp. 695–695. DOI: 10.1038/427695a.

[10] J. Kremheller, A.-T. Vuong, B. A. Schrefler, and W. A. Wall. “An approach for vascular tumor growth based on a hybrid embedded/homogenized treatment of the vasculature within a multiphase porous medium model”. In: *International Journal for Numerical Methods in Biomedical Engineering* (2019), e3253. DOI: 10.1002/cnm.3253.

[11] V. Vavourakis, T. Stylianopoulos, and P. A. Wijeratne. “In-silico dynamic analysis of cytotoxic drug administration to solid tumours: Effect of binding affinity and vessel permeability”. In: *PLOS Computational Biology* 14.10 (2018). Ed. by A. D. McCulloch, e1006460. DOI: 10.1371/journal.pcbi.1006460.

[12] B. Müller, S. Lang, M. Dominietto, M. Rudin, G. Schulz, H. Deyhle, M. Germann, F. Pfeiffer, C. David, and T. Weitkamp. “High-resolution tomographic imaging of microvessels”. In: *Developments in X-Ray Tomography VI* 7078.August (2008), 70780B. DOI: 10.1117/12.794157.

[13] T. Stylianopoulos and R. K. Jain. “Combining two strategies to improve perfusion and drug delivery in solid tumors”. In: *Proceedings of the National Academy of Sciences of the United States of America* 110.46 (2013), pp. 18632–7. DOI: 10.1073/pnas.1318415110.

[14] L. T. Baxter and R. K. Jain. “Transport of fluid and macromolecules in tumors. II. Role of heterogeneous perfusion and lymphatics”. In: *Microvascular Research* 40.2 (1990), pp. 246–263. DOI: 10.1016/0026-2862(90)90023-K.

[15] R. W. Lewis and B. A. Schrefler. *The finite element method in the static and dynamic deformation and consolidation of porous media*. John Wiley, 1998.

[16] B. A. Lewis, R. W. Schrefler. *The finite element method in the deformation and consolidation of porous media*. Wiley, 1987.

[17] W. A. Wall et al. *BACI: A multiphysics simulation environment*. Technical report, Institute for Computational Mechanics, Technical University of Munich, 2019.

[18] L. Cattaneo and P. Zunino. "A computational model of drug delivery through microcirculation to compare different tumor treatments". In: *International Journal for Numerical Methods in Biomedical Engineering* 30.11 (2014), pp. 1347–1371. DOI: 10.1002/cnm.2661.

[19] M. Wu, H. B. Frieboes, S. R. McDougall, M. A. Chaplain, V. Cristini, and J. Lowengrub. "The effect of interstitial pressure on tumor growth: Coupling with the blood and lymphatic vascular systems". In: *Journal of Theoretical Biology* 320 (2013), pp. 131–151. DOI: 10.1016/J.JTBI.2012.11.031.

[20] L. T. Baxter and R. K. Jain. "Transport of fluid and macromolecules in tumors. I. Role of interstitial pressure and convection". In: *Microvascular Research* 37.1 (1989), pp. 77–104. DOI: 10.1016/0026-2862(89)90074-5.

[21] R. Santagiuliana, M. Ferrari, and B. A. Schrefler. "Simulation of angiogenesis in a multiphase tumor growth model". In: *Computer Methods in Applied Mechanics and Engineering* 304 (2016), pp. 197–216. DOI: 10.1016/j.cma.2016.02.022.

[22] A. S. Popel. "Theory of oxygen transport to tissue". In: *Critical Reviews in Biomedical Engineering* 17.3 (1989), pp. 257–321.

[23] M. Welter, T. Fredrich, H. Rinneberg, and H. Rieger. "Computational Model for Tumor Oxygenation Applied to Clinical Data on Breast Tumor Hemoglobin Concentrations Suggests Vascular Dilatation and Compression". In: *PLOS ONE* 11.8 (2016), e0161267. DOI: 10.1371/journal.pone.0161267.

[24] D. Goldman. "Theoretical models of microvascular oxygen transport to tissue". In: *Microcirculation* 15.8 (2008), pp. 795–811. DOI: 10.1080/10739680801938289.
