# Supplementary material for: Extension of a multiphase tumour growth model to study nanoparticle delivery to solid tumours
Source: PLoS One. 2020 Feb 5;15(2):e0228443. doi: 10.1371/journal.pone.0228443 (PMC7001947; doi:10.1371/journal.pone.0228443)
Supplement: Attachment — Submitted filename: Answers.pdf [file pone.0228443.s002.pdf]

Response to Review #1:

The authors would like to thank the reviewer for his/her comments that helped to improve the manuscript. In the following all points of the reviewer are addressed, where first the reviewers comment is repeated in *italic* and then replied to in blue color. Changes to the manuscript that have been made are marked in blue color in the revised version. The removal of text at certain locations is highlighted with ~~red~~ color in the revised manuscript.

The authors have previously developed a multiphase model for tumor growth. Here they extend the model to examine the factors influencing nanoparticle delivery to tumors. The main conclusion is that only very small nanoparticles, for which transport is diffusion dominated, can reach the entire tumor. The effects of varying key parameters describing wall permeability to nanoparticles are investigated. The analysis is carefully done and clearly presented, and the results appear plausible. The approach used is more elaborate than would be needed to establish these results. For example, the model is used to simulate the dynamics of tumor growth, but the nanoparticle transport simulations are done for the configuration at 160 hours, which could perhaps have been established based on previously published observations and/or simulations. However, this does not invalidate the results obtained. The strengths and limitations of the approach are appropriately discussed. Overall, while the work does not lead to striking new insights, it is a valid contribution to the literature on this topic, providing quantitative information about the factors influencing nanoparticle delivery to tumors.

We appreciate the reviewer's positive feedback and are happy to comment on his/her remarks.
We agree with the reviewer that the full tumor growth model is only used as a precursor to generate a physically plausible initial configuration for the nanoparticle transport study. In response to the comments of both reviewers we have therefore decided to move the equations and most of the model formulation into an appendix to streamline the paper and to convey its main message more clearly. Only the terms influencing nanoparticle transport are now elaborated in the main document.
The reviewer is also correct that equivalent results could have been generated based on experimental data or previous simulations. We want to emphasize that we have tried to carefully check the plausibility of the results obtained with the growth compartment of the model in Section 3.1 to ensure that we start the drug delivery based on a physically meaningful solution. This justifies leaving this analysis in the main document. Nevertheless, we also believe that it has several benefits to study drug delivery in combination with a tumor growth model in the background (which have not been fully exploited in this paper because we have not yet included any uptake and killing mechanism of drugs). Also we consider this as a first step to complete the model for the evaluation of drug efficiency. This requires the introduction of drug uptake and of a killing mechanism, while the growth model is already there.
Already at this step, it allows generating many physically plausible configurations easily and reliably if the tumor growth model is well calibrated and validated. Furthermore, the effect of periodic treatments with intermittent tumor growth can be assessed. It also allows the study of novel therapies, such as vascular normalization followed by treatment with a conventional drug. We have added a short discussion in the conclusion section.

Response to Review #2:

The authors would like to thank the reviewer for his/her comments that helped to improve the manuscript. In the following all points of the reviewer are addressed, where first the reviewers comment is repeated in *italic* and then replied to in blue color. Changes to the manuscript that have been made are marked in blue color in the revised version. The removal of text at certain locations is highlighted with ~~red~~ color in the revised manuscript.

Overall, I found this paper interesting, especially the nanoparticle transport study. I think the authors' systematic study of the different transport barriers and the sensitivity of the drug transport to different parameters is well designed and satisfies the Plos One criteria for publication. There are a few points that I would like the authors to address and/or discuss in their manuscript.

We appreciate the reviewer's positive feedback and are happy to comment on his/her remarks.

1. First, the storyline of the paper is built around the extension of a multiphase tumor growth model to study nanoparticle delivery, but the role of the tumor model is minor. All the results and conclusions are focused on the nanoparticle transport. As far as I understand the results in Fig. 6 where produced only with the nanoparticle transport compartment of the model (the tumor dynamics is frozen, right?). If all of these assumptions are correct, I think there is a lot of information in the paper that deviates the reader from the main message, which comes across more weakly.

We agree with the reviewer that the full tumour growth model is only used as a precursor to generate a physically plausible initial configuration for the nanoparticle transport study. In response to the comments of both reviewers we have therefore decided to move the equations and most of the model formulation into an appendix to streamline the paper and to convey its main message more clearly. Only the terms influencing nanoparticle transport are now elaborated in the main document.
Tumour dynamics while calculating the nanoparticle distributions in Section 3.2 is not frozen, but due to the difference in time scales it does not influence the results during the short time period which is simulated as correctly pointed out by the reviewer. This information has been added to Section 3.2. We want to emphasize that we have tried to carefully check the plausibility of the results obtained with the growth compartment of the model in Section 3.1 to ensure that we start the drug delivery based on a physically meaningful solution. This justifies leaving this analysis in the main document. Nevertheless, we also believe that it has several benefits to study drug delivery in combination with a tumor growth model in the background (which have not been fully exploited in this paper because we have not yet included any uptake and killing mechanism of drugs). Also we consider this as a first step to complete the model for the evaluation of drug efficiency. This requires the introduction of drug uptake and of a killing mechanism, while the growth model is already there.
Already at this step, it allows generating many physically plausible configurations easily and reliably if the tumor growth model is well calibrated and validated. Furthermore, the effect of periodic treatments with intermittent tumor growth can be assessed. It also allows the study of novel therapies, such as vascular normalization followed by treatment with a conventional drug. We have added a short discussion in the conclusion section.

2. There is a lot of notation in the paper. It is difficult to keep track of the symbols. A table with all the notation would help.

We agree with the reviewer and thus have put a nomenclature into the supporting information of the paper.

3. In the discussion of Fig. 4, how is the tumor radius defined? (the tumor is not circular). Also how is 'the dege of the tumor' defined? As far as I can see all variables are continuous across the tumor boundary.

Yes, the variables are continuous across the tumor boundary. The edge of the tumor is defined as $S^t = 0.1$. Based on this, the radius is estimated as the mean value of the Euclidean distance of the edge to the center of the tumor. We also added this to the paper.

4. The images look blurry, to the point that I had a hard time reading the text on them.

We would like to apologize for this inconvenience. We believe that this has to do with the submission system. Actually, we have provided high-resolution *.tiff pictures, which can also be downloaded when clicking on the links above the respective figures. We will make sure that high-quality pictures will be used for the final version.
